# Study on smoke blocking and thermal radiation attenuation by water curtain in tunnel fire

**DOI:** 10.1038/s41598-023-27437-2

**Published:** 2023-01-05

**Authors:** Yinuo Chen, Jinzhang Jia, Guangbo Che, Zhiheng Zhu, Zhiyuan Shen, Yumo Wu

**Affiliations:** 1 School of Safety Science and Engineering, Liaoning Technical University, Fuxin, 123000 Liaoning China; 2 Key Laboratory of Mine Thermal Disaster and Prevention, Ministry of Education, Huludao, 125000 Liaoning China; 3 Key Laboratory of Preparation and Applications of Environmental Friendly Materials (Jilin Normal University), Ministry of Education, Changchun, 130103 People’s Republic of China

**Keywords:** Energy science and technology, Engineering

## Abstract

A 1:10 scale model tunnel with a length, height and width of 9 m, 0.6 m and 0.8 m, respectively, was set up in this paper. A water curtain system was installed in the model to investigate the effect of water curtain systems on smoke flow and heat propagation. A reduced-scale experimental and theoretical study was carried out by varying the heat release rate of the fire source, the water curtain pressure, and the number of water curtain rows. A series of tests were carried out for various setups to quantify each mechanism of interaction between the water mist and hot smoke, to propose a method for qualitatively analysing water curtain systems blocking the propagation of heat radiation and the flow of smoke from combustion, and to propose a method for predicting heat fluxes. The study found that the pressure of the water curtain, the number of rows, and the heat release rate of the fire source all had an effect on the smoke blocking effect of the water curtain system. This effect decreased as the heat release rate of the fire source increased and increased significantly with the pressure of the water curtain and the number of rows. The smoke blocking effect was quantified using conservation of momentum by establishing a dimensionless parameter *R* to represent the ratio of water curtain momentum to smoke momentum, as well as the ratio of heat flux before and after the water curtain to represent the smoke blocking capacity $\delta$ of the water curtain. The smoke blockage rate $\delta$ ranges between 40 and 75%, and the smoke blockage rate increases as the momentum *R* increases. Finally, in tunnel fires, a predictive model for the attenuation of heat radiation by water curtains has been developed, providing theoretical support for the quantitative study of the smoke and thermal blockage effects of water curtains, which is beneficial to the protection of human life in confined spaces.

## Introduction

A tunnel has the characteristics of a high volume of traffic, long and narrow shape, and significant environmental impact. In the event of a fire, tunnel visibility is low, fire-fighting and rescue operations are challenging, smoke production is high, smoke toxicity is high, ambient temperature is high, and the fire's intensity and rate of development are all high^1–5^. The high-temperature environment brought by tunnel fires and the toxic smoke produced by combustion cause serious consequences, such as damage to vehicles and equipment in the tunnel, and the death and injury of people. As a result, regulating high-temperature smoke and extinguishing or suppressing tunnel flames are essential to preventing tunnel fires.

The majority of early studies in the field of tunnel fire safety concentrated on fire-induced smoke flow, smoke stratification, and smoke temperature^6–9^. The water curtain system will be employed to suppress the fire and control the flow of smoke and the spread of heat due to the unique ventilation and traffic conditions in the tight section and long tunnel structure. Water mist has the inherent features of high efficiency, minimal destruction, and being non-polluting. The water curtain system, which emits water mist, is also widely considered a clean and effective fire suppression device^10^. Scholars worldwide have examined the use of water curtains in tunnels, with the majority of the research beginning with both experimental and numerical simulations. In experimental studies^11–15^, it was found that the activation of the water curtain system to mix the smoke around it with air was effective in attenuating thermal radiation and blocking the propagation of toxic and harmful gases through reduced size or full scale experiments. The activation of the water mist system has a powerful cooling impact and significantly lowers the temperature inside the tunnel, even though it does not completely stop the spread of combustion smoke. In numerical simulations^16,17^ used FDS to simulate water spray tests in reduced- or full-scale tunnel fires. It was found that the movement between the spray and the smoke, with the apparent upwards movement in the area between the sprays being caused by the spray-induced jets hitting the ground, acted as a barrier to prevent the smoke from spreading downstream of the sprays.

Despite the fact that FDS simulations have been used extensively in fire research, many articles show that computational fluid dynamics (CFD) tools are useful for understanding how fire and spray interact^18–20^. However, the smoke barrier insulation mechanism of the interaction between fire and water spray has not yet been fully understood due to a lack of experimental data and instrumentation issues. Li et al.^21^ obtained values for the momentum ratio R through a 1:3 scale experiment and discovered that R can characterize the effect of smoke obstruction under a water curtain system. They quantified the relationship between the kinetic energy of water jets and combustion smoke through the law of conservation of mass to qualitatively characterize the effect of combustion smoke obstruction by a water curtain. In terms of mass flow, Tanaka et al.^22^ discovered that the law of conservation of mass can be used to quantify the mass flow ratio of smoke from a volume of combustion smoke, as well as through analysis of combustion smoke gas composition, primarily using CO_2_ to represent smoke mass flow, combined with the combustion gas composition equation of White et al.^23,24^ to derive the CO_2_ fraction, i.e., the smoke blocking efficiency.

Scholars in the preceding works focused on determining the dispersion of smoke, temperature, and so on induced by water curtain systems utilizing reduced-size experiments and CFD methodologies. Fewer researchers have quantified the effect of water curtains on smoke blockage, so we present a detailed experimental analysis of the effect of water spray and fire interaction on smoke blockage in tunnel fires using small-scale experiments in this paper. Second, using the law of conservation of kinetic energy, we quantify the momentum ratio and thermal radiation attenuation to characterize the smoke barrier efficiency, and we examine the relationship between the momentum ratio and thermal radiation attenuation once more. Finally, based on the parameters having an impact on fire, a predictive model for the attenuation of thermal radiation from water curtains in tunnel fires was developed, with significant implications for fire prevention and control in long and narrow spaces, as well as personnel protection and rescue.

## Experimental setup

In this study, the 1:10 scale for this model tunnel would correspond to an actual tunnel size of 8 m in width, 6 m in height, and 90 m in length, with the model employing a width of 0.8 m, a height of 0.6 m, and a length of 9 m, built inside the laboratory. The model scaling law based on Froude's criterion is shown in Table 1^25–27^.

**Table 1:** Scaling relationships for characteristic parameters.

Unit	Scaling (*S* = scale ratio)
Length (m)	*L*_*full*_/*L*_*reduce*_ = *S*
Heat release rate (kW)	Q _*full*_/Q_*reduce*_ = *S*^*5/2*^
Water flow (L/min)	*q*_*w full*_/*q*_*w reduce*_ = *S*^*5/2*^
Droplet size (μm)	*d *_*full*_/*d *_*reduce*_ = *S*^*1/2*^
Time (s)	*t *_*full*_/*t*_*reduce*_ = *S*^*1/2*^
Longitudinal wind speed (m/s)	*V *_*full*_/*V*_*reduce*_ = *S*^*1/2*^
Temperature (°C)	T _*full*_/*T*_*reduce*_ = *S*^*0*^ = 1

### Model tunnels

**Figure 1:**
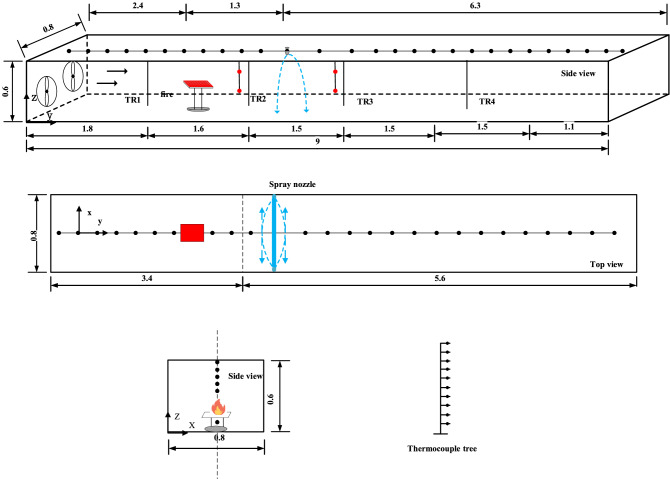
A sketch view of the model tunnels.

Figure 1 illustrates a small-scale model tunnel (1/10th the actual tunnel size). This scale has been utilized in various tunnel fire experiments^15,22,28^. The experimental tunnel is made up of nine small units, each measuring 1.0 m in length. The tunnel's main body is built of a white steel frame, its ceiling, bottom, and rear sidewalls are made of 12 mm thick fire-resistant gypsum board, and the front sidewalls are made of 10 mm thick laminated fireproof glass, making it easy to observe and record the smoke flow process in the passage. To avoid the influence of the frame structure on the movement of the smoke flow from the fire, the panels and fireproof glass are set inside the steel frame and secured with bolts, as well as the application of fireproof adhesive to ensure the accompanying air tightness requirement for the tunnel. An axial fan is installed on the left side of the tunnel to provide longitudinal uniform air flow, and a fuel tray is installed inside the tunnel at a vertical elevation of 10 cm above ground level.

**Figure 2:**
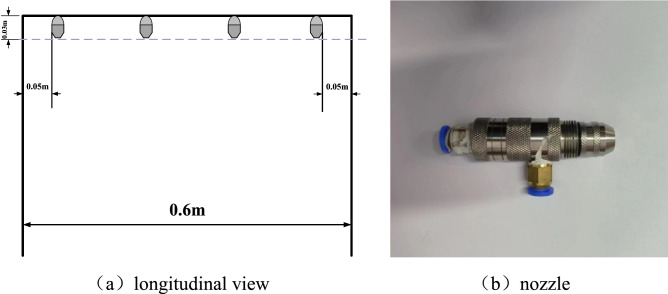
Schematic diagram of nozzle setup.

Figure 2 depicts a row of four conical spray nozzles forming a water curtain 1.8 m downstream from the fire source, directly above the tunnel cross section. In “Bucket test” section, bucket tests were performed to explain the details of the water spray droplet size distribution and to measure these jet characteristics using the same experimental setup as the spray mass flux distribution measurements.

### Measurements

The experiments were carried out with a methanol fire source, and the fire heat release rate *Q* was estimated by measuring the residual mass after combustion with an electronic balance and computing the mass loss rate *m*^29^. Then, based on the combustion value $\eta$ and combustion efficiency $\Delta H$, *Q* was computed as

1 $$Q = \eta \cdot \Delta H \cdot {\text{m}}$$

where $\Delta H$ is the combustion efficiency factor, $\eta$ is the combustible material's calorific value in kJ/g, and *m* is the combustible material's mass burning rate. Methanol has a calorific value of combustion of 19.93 kJ/g and a combustion efficiency of 0.9^30^.

This study was performed using four different types of oil pans with side lengths ranging from 10 to 13 to 16 to 20 cm. The study was carried out using an electronic balance with a precision of 0.01 g. For four experiments utilizing different oil pans, the heat release rates of the fire experiments were calculated to be 4.6, 7.4, 9.8, and 15.3 kW. The temperature tests were performed with 2 mm diameter K-type armoured thermocouples, model WRNK-191, with a measurement temperature range of 0 to 1100 °C. To measure the vertical temperature, four rows of thermocouple trees were planted in the tunnel at 1.8, 3.2, 5.6, and 7.4 m. Taking into consideration the accuracy of the thermocouples and the standard deviation of the mean temperature, the uncertainty of the mean temperature during the quasi-steady state ranged from 0.1 to 3.8 °C. The variability in the temperature measurement of the water-wetted thermocouple can be overlooked in the experiment since the thermocouple near the nozzle is unaffected by water. Radiant heat flow meters are installed at 3 and 4.6 m to measure the radiant heat flow before and after the water curtain system. The temperature during the experiments was approximately 21.2 °C. To obtain average values, each fire experiment was performed in triplicate under each set of experimental conditions^27^.

### Summary of test settings

The experimental conditions change depending on the rate of heat release from the fire source, as well as the water spray pressure and number of rows in the water curtain system. To the greatest extent practicable, the beginning and boundary conditions are regulated identically in all circumstances. Because the tunnel model was built indoors, the ambient temperature was kept at approximately 20 °C with a maximum fluctuation of 5%. The specific experimental setup is shown in Table 2.

**Table 2:** Summary of settings in different fire tests.

Case	HRR (kW)	Water curtain row	Working pressure (Mpa)	Ambient temperature (°C)
T_1_–T_4_	4.6	1	0.2,0.4,0.6,0.8	21.1
T_5_–T_8_		2	0.2,0.4,0.6,0.8	20.7
T_9_–T_12_		3	0.2,0.4,0.6,0.8	20.1
T_13_–T_16_	7.4	1	0.2,0.4,0.6,0.8	22.3
T_17_–T_20_		2	0.2,0.4,0.6,0.8	21.8
T_20_–T_24_		3	0.2,0.4,0.6,0.8	21.4
T_25_–T_28_	9.7	1	0.2,0.4,0.6,0.8	22.9
T_29_–T_32_		2	0.2,0.4,0.6,0.8	22.1
T_33_–T_36_		3	0.2,0.4,0.6,0.8	21.4
T_37_–T_40_	15.3	1	0.2,0.4,0.6,0.8	23.6
T_41_–T_44_		2	0.2,0.4,0.6,0.8	23.1
T_45_–T_48_		3	0.2,0.4,0.6,0.8	22.5

## Bucket test

**Figure 3:**
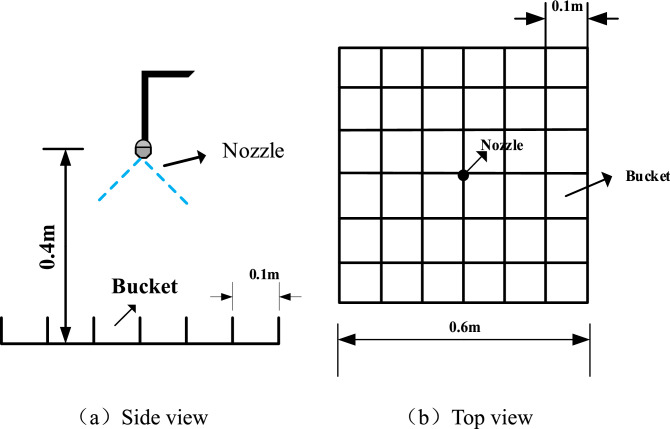
Schematic diagram of the bucket experimental setup: (**a**) side view; (**b**) top view.

This study employs bucket testing to measure water flux on the nozzles of the water curtain system, as shown in Fig. 3. In this test, 6 rows of measuring buckets, 6 in each row, are set beneath the nozzle, the buckets are made of 3 mm thick PMMA with dimensions of 10 cm × 10 cm, the vertical distance from the nozzle to the ground is 0.4 m, and the water mist collection duration is 3 min for repeated tests. The spray is initially inconsistent, but since this phenomenon only lasts approximately 3 s after spray activation, it is insignificant throughout the experiment. The experimental circumstances for the bucket test were identical to those of the tunnel fire experiment, and Liu et al.^31^ measured spray mass flow using a similar technique.

**Figure 4:**
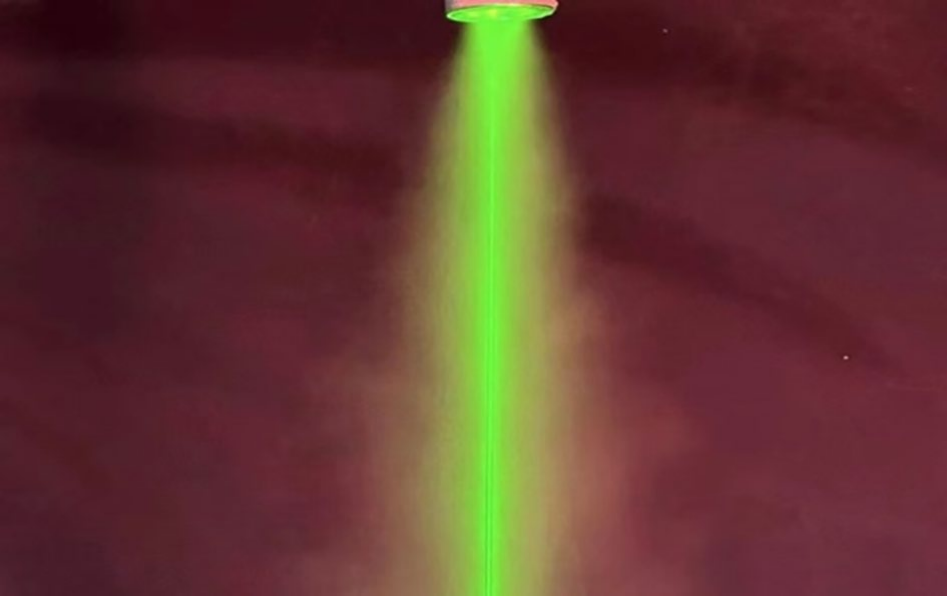
Water mist image of the experimental sprinkler.

Figure 4 depicts a conical area of fine water spray from an experimental nozzle with a 0.12 mm aperture. At the same time, the water flow is measured using a measuring ring. In this case, a nozzle pressure of 0.6 MPa is utilized to determine the distribution of water droplet diameters using a laser particle size meter and to detect the relative energy scattered by the water droplets on a photodetector.

**Figure 5:**
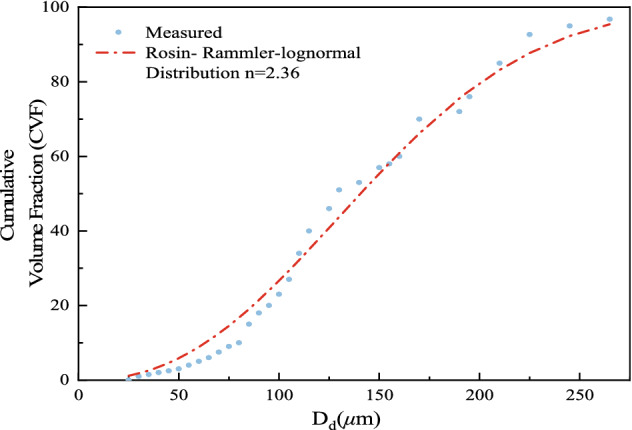
Droplet size distributions for 0.6 MPa.

The cumulative volume fraction (CVF) curve of the droplets, as shown in Fig. 5, corresponds nicely with the Rosin‒Rammler curve^32,33^.

2 $${\text{CVF}}( {D_{v} }) = 1 - \exp^{{ - 0.693\left(d/{D_{v50}} \right){n}}}$$

*D*_v_50 is the median particle size (50% of the total droplet volume consists of all droplet diameters from zero to this value), and n is the distribution coefficient. In this study, the parameter n = 2.36 was determined from the comparison in Fig. 5.

Using the following formula, the water flux ${\dot{\text{m}}}_{w}^{\prime\prime}$ (L/min/m^2^) is calculated in each test bucket.

3 $$\dot{m}_{w}^{\prime\prime} = \frac{{M_{bucket} }}{{A_{bucket} \Delta t_{bucket} }}$$

where $M_{{{\text{bucket}}}}$ is the approximate mass of water received by the test bucket, kg; $A_{{{\text{bucket}}}}$ is the open surface area of the top of the bucket, m^2^; and $\Delta t_{bucket}$ is the spraying duration, min.

**Figure 6:**
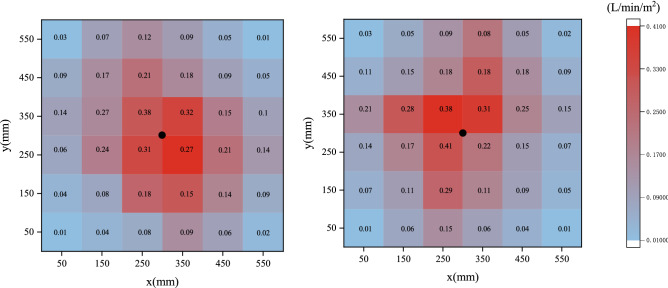
Water flux density distribution results for0.6 MPa.

Figure 6 depicts the bucket test water flux density distribution results. The total water flux collected in the bucket was approximately 1–3% lower than the water flux from the nozzle (0.35 L/min), as some water dropped on the ground outside the range of the measuring bucket due to space limitations at the experimental site. It should also be noted that the nozzle's internal angle is difficult to adjust to a direction perpendicular to the ground, resulting in maximum water flux density readings that are not in the same place each time they are measured.

Experiments were performed with four nozzle pressures of 0.2 MPa, 0.4 MPa, 0.6 MPa, and 0.8 MPa, and the results may be found in Table 3 “Nozzle Parameter Characteristics”.

**Table 3:** Nozzle pressure characteristics parameters.

Nozzle working pressure (Mpa)	Atomisation cone angle (°)	Nozzle flow rate (L/min)	Droplet diameter (μm)	Distribution coefficient n
0.2	65	1.41	256	2.96
0.4	71	1.62	234	2.59
0.6	78	2.01	219	2.36
0.8	84	2.34	172	2.17

## Results and discussion

### Effect of the water curtain on the temperature in the tunnel

Temperature measurements were utilized to evaluate the smoke blocking effect of the water curtain and to qualitatively identify the fraction of smoke entrained and released. The temperature of smoke moving downwards as generated by a water curtain system may be experimentally detected by a waterproof thermocouple, and the temperature of this smoke produced by water mist can be traced by an increase in temperature in the lower space. Taking into account the accuracy of the thermocouple and the standard deviation of the mean temperature into account, the uncertainty in the mean temperature during the quasi-steady state is in the range of 0.1–2 °C. The uncertainty of temperature measurement of the water-wetted thermocouple can be ignored in this study since the thermocouple near the nozzle is not affected by water.

#### Temperature variation at varying heat release rates

**Figure 7:**
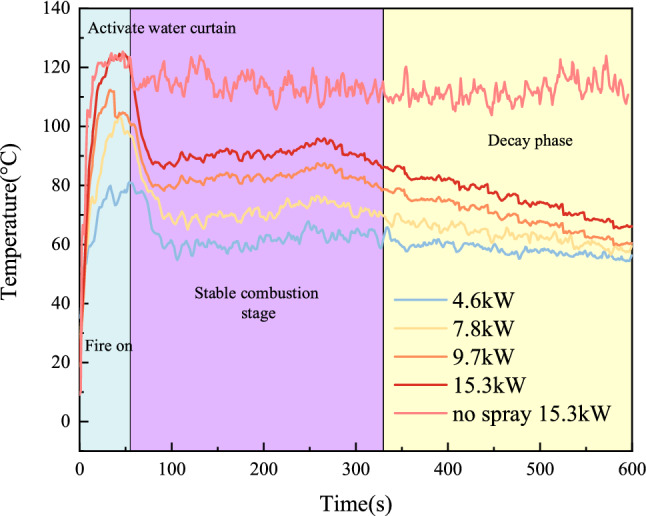
Comparisons of temperature rise profiles under different heat release rates.

Figure 7 depicts the progression of the tunnel ceiling centerline temperature 20 cm below the surface in a cross section 1.6 m downstream of the fire source. All other testing conditions were identical, and the heat release rates for the fire were 4.6 kW, 7.8 kW, 9.7 kW, and 15.3 kW. The gas temperature drops rapidly from its peak when the water curtain system is activated and remains practically constant between 180 and 330 s after ignition, a time of stable combustion for the fire. After 330 s an overall declining trend in temperature is seen. The following average experimental data are for the quasi-steady state period between 180 and 330 s after ignition.

**Figure 8:**
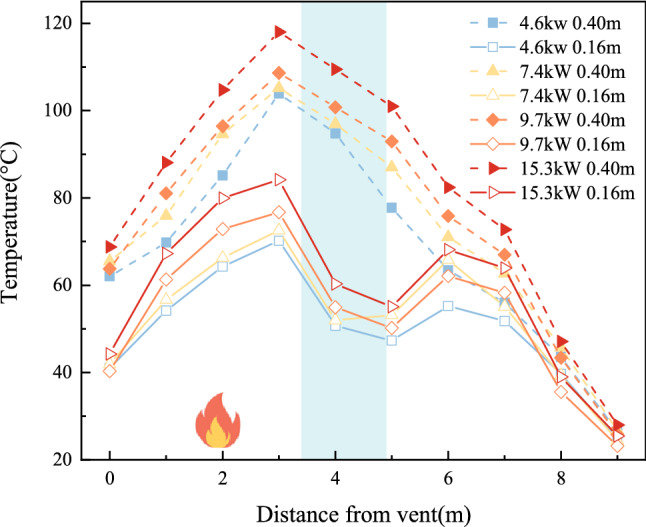
Distribution of average temperature at different heights of the tunnel under different heat release rates.

Figure 8 depicts the average temperature distribution at tunnel heights of 0.4 m and 0.16 m for experiments T_12_, T_24_, T_36_, and T_48_ to study the influence of varying heat release rates on the temperature within the water tunnel. The heat release rates of the fire source for the eight experimental groups were 4.6 kW, 7.8 kW, 9.7 kW, and 15.3 kW, while all other experimental conditions remained constant.

Figure 8 shows the temperature change curve at z = 0.4 m and z = 0.16 m along the longitudinal centreline of the tunnel. The temperature first increases and then decreases with increasing distance from the vent. This is because the area from the vent to the fire source is close to the fire source, the ventilation and heat dissipation effect is slow, and the temperature rises rapidly. The temperature near the fire source reaches its peak value. After cooling in the water curtain area, the absorbed heat increases, and the temperature drops as a whole. The temperature under the tunnel roof decreases. With an increase in the power of the fire, the heat generated increases, and more heat is absorbed by the water mist particles, leading to a better cooling effect.

However, at the bottom plate position of the tunnel (z = 0.16 m), the temperature will rise near the water curtain, which can be explained by the smoke logging theory. The higher the temperature of the flue gas layer, the more stable it is under the action of water curtain spraying because the high-temperature flue gas is often subject to stronger buoyancy. With the operating conditions of the water mist system held constant, the difference between the temperature rise before and after the water curtain at the tunnel floor is greater, which indicates that the settling of the smoke is more prominent, and thus that most of the hot smoke is absorbed by the water mist. The reduction of smoke by means of the water mist indicates a better smoke suppression effect of the water mist system. When the power of the fire is small, the smoke suppression effect of the water curtain fog system is more obvious.

#### Temperature variation at varying row counts

Each of the eight experimental groups had one, two, or three rows of water curtain, with all other experimental conditions remaining constant.

**Figure 9:**
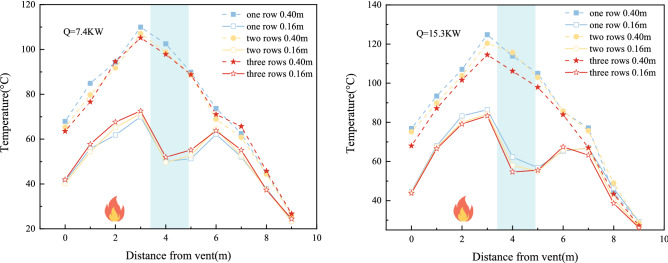
Distribution of average temperatures at different heights in the tunnel with different rows.

Figure 9 shows the temperature distribution at z = 0.4 m and z = 0.16 m in the experimental tunnel. As shown in the figure, as the number of water curtain rows increases, the temperature at the two heights above the roadway shows a greater decreasing trend. This is because the more nozzles that are opened, the greater the amount of water spray that will be generated, and the volume concentration of water mist droplets will increase. This will result in stronger cooling and absorption. However, at z = 0.16 m, although the cooling effect of water spray is stronger when more rows have their nozzles open, the temperature before and after the water curtain at this height still shows an upwards trend with the increase in the number of rows with open nozzles. At the same water pressure, the more nozzle rows that are opened, the more obvious is the temperature rise effect under the tunnel. In addition, with more open nozzle rows, the greater the fire source heat release rate and the more obvious the temperature rise effect under the tunnel. This indicates that the more prevalent the settling of the smoke, the greater the amount of hot smoke absorbed by the water mist. This leads to the reduction of smoke passing through the water mist, evidencing a better smoke suppression effect of the water mist.

#### Temperature variation at varying pressures

To investigate the effect of different nozzle pressures on tunnel temperature, the nozzle pressures in the eight experimental groups were 0.2 MPa, 0.4 MPa, 0.6 MPa, and 0.8 MPa, while the other experimental conditions remained constant.

**Figure 10:**
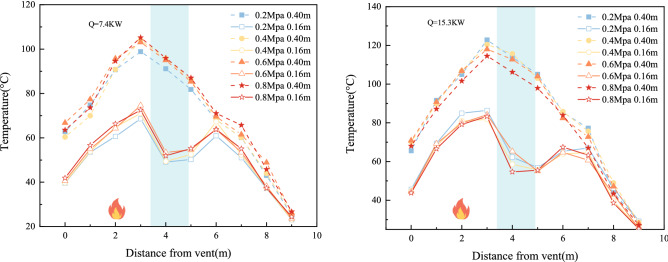
Distribution of average temperatures at different heights of the tunnel at different water pressures.

Figure 10 shows the temperature distribution at z = 0.4 m and z = 0.16 m in the experimental tunnel. The overall temperature in the tunnel decreases with increasing nozzle pressure. This is because the higher the water pressure, the smaller the water particles generated, and the greater the number of water particles. A greater number of smaller water particles will show a stronger cooling effect. At the same time, the higher the pressure is, the greater the water spray flow is, and the stronger the absorption effect on fire heat smoke is, resulting in a positive correlation between temperature attenuation and water spray pressure. The temperature at z = 0.16 m shows an upwards trend with increasing spray pressure, which is due to the increase in the area of high-temperature smoke entrained by the spray after an increase in pressure at the nozzle. With the same number of water curtain rows, the greater the water pressure, the greater the difference in temperature rise before and after the water curtain in the tunnel. At the same time, the greater the power of the fire is, the greater the difference in temperature rise, which indicates that the more prevalent the settling of the smoke, which further indicates that less smoke passes through the water mist system, which indicates a better smoke suppression effect of the water mist system.

#### Longitudinal temperature variation at varying distances

**Figure 11:**
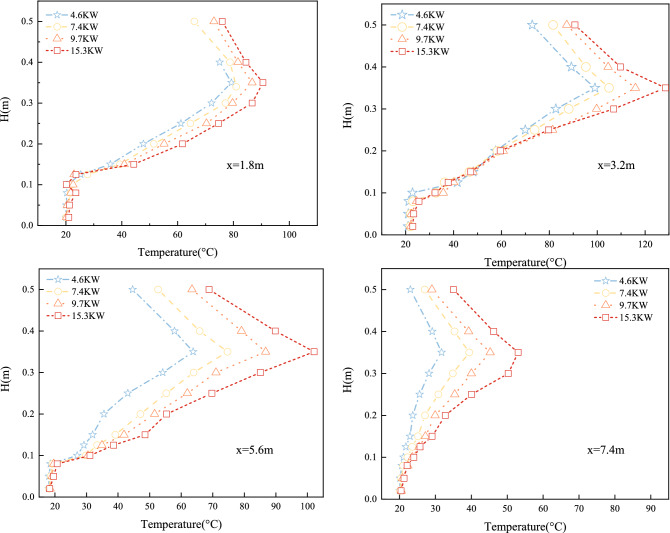
Time-averaged (150–250 s) temperature variation curve with height.

The analysis of the water curtain pressure, number of rows, and heat release rate of the fire source revealed a temperature difference between the ceiling and floor of the tunnel, so the longitudinal temperature variation in the tunnel was investigated next. Figure 11 depicts x = 1.8 m, x = 3.2 m, x = 5.6 m, and x = 7.4 m with experimental fire source heat release rates of 4.6 kW, 7.8 kW, 9.7 kW, and 15.3 kW. All other experimental conditions remained constant.

Figure 11 depicts the temperature distribution of the four thermocouple trees at various fire scales. The vertical axes of the coordinates represent the tunnel height, while the horizontal axes represent the temperature values. Although the water curtain significantly reduces the temperature rise on the exit side, the temperature rise remains significant due to the hot smoke generated by the fire. The airflow travels from left to right towards the water mist. The momentum of the water being blown away by the longitudinal air flow attenuates the smoke blocking effect of the water curtain and causes hot smoke to escape from the water jet area. Although the location of the thermocouple trees varies, the overall temperature change in the tunnel can be seen, and the change in thickness of the smoke layer is positively correlated with the fire heat release rate. The larger the fire size, the more obvious is the greater thickness of the smoke layer stratification, and the temperature stratification at the fire source at x = 3.2 m is the clearest. This is the hot smoke from the fire above the tunnel in the downwards flow duplex.

### Effect of the water curtain on smoke blockage in the tunnel

Water curtain systems are installed in tunnels to control both the spread of smoke within the fire zone and thermal radiation damage to personnel as much as possible. The effect of water curtain systems on temperature changes in tunnels was described in “Effect of the water curtain on the temperature in the tunnel” section. It can be seen that the addition of fine water mist can reduce the temperature in the tunnel, especially before and after the water curtain system, which will increase the smoke settling situation. This will reduce the smoke entering the water mist system and create a smoke blockage phenomenon. Thus, in this paper, a study was conducted to quantify the smoke blocking effect, and a qualitative method is also presented.

#### Interaction of smoke and water curtain

**Figure 12:**
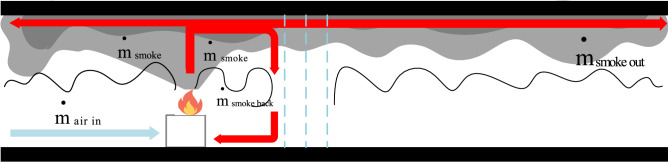
The movement of air and smoke after the activation of the water curtain system.

Figure 12 depicts the movement of smoke from the fire caused by the water curtain system. The large amount of smoke generated in the fire area is divided into two parts: part of the smoke moves along the tunnel ceiling through the water curtain system outwards and part of the smoke moves along the ceiling outflow process by the influence of water mist on the tunnel floor entrainment. the phenomenon of smoke roll sucking, and roll sucking back into the ceiling jet. The tunnel in this study is open at both ends, with a vent on the left end and a water curtain system on the right. Based on the law of conservation of momentum, we can derive the following equation assuming a confined space between the water curtain and the vent.

4 $$M_{out} = M_{{{\text{smoke}}}} - M_{back}$$

where *M*_*smoke*_ represents the total momentum of the smoke produced by the fire; *M*_*back*_ represents the momentum of the returned smoke beneath the water mist; and *M*_*out*_ represents the momentum of the smoke as it passes through the water curtain system.

Many factors influence the smoke control characteristics of the water curtain system, including the design parameters of the water curtain system, rate of heat release from the fire source, and tunnel size. To quantify the effect of the water curtain system on smoke, the momentum ratio R is defined as the ratio of the vertically downwards water curtain spray momentum to the total momentum of the smoke produced by the fire, as shown in the following equation.

5 $$R = \frac{{M_{{{\text{water}}}} }}{{M_{{{\text{smoke}}}} }} = \frac{{\rho_{{\text{w}}} A_{w} v_{w}^{2} }}{{\rho_{s} A_{s} v_{s}^{2} }}$$

where *ρ*_*w*_ is the density of water, kg/m^3^; *v*_*w*_ is the velocity of the water curtain jet, m/s; and *ρ*_*s*_ is the smoke density in the fire area, kg/m^3^. *A*_s_ is the smoke inlet area, m^2^, whose value is the product of the tunnel width y and tunnel height z; *v*_*s*_ is the smoke spread velocity of the fire area, m/s.

By extrapolating the smoke spread time from full-scale experiments, Hu et al.^34–37^ determined that the smoke spread velocity in a tunnel decreases linearly with distance from the fire source. We can extrapolate the speed of the smoke front in the experiments by video recording the speed of smoke spread or by the time difference of the thermocouple temperature rise. The water jet velocity is set to the average flow velocity of water particles *v*_w_ (m/s), which is assumed to be half the initial velocity of particles ejected from the nozzle^38^.

6 $$v_{w} = \frac{1}{2}{{{\dot{\text{m}}}_{{\text{w}}} }/{\left( {{\text{s}} \cdot \rho_{{\text{w}}} } \right)}}$$

where *s* is the area of the nozzle orifice, m^2^. When *R*>>1, the momentum of the water curtain exceeds the momentum of the smoke, indicating that the water curtain system can impede smoke spread.

#### Interaction of heat and the water curtain

The smoke blocking performance of the tunnel water curtain wall reduces heat transmission, thus attenuating high-temperature smoke transfer. With water curtains set in front and behind the heat radiation mark to measure heat flux, a more intuitive reflection of a series of changes in the tunnel heat may be seen. As a result, thermal radiation attenuation efficiency can be expressed as follows.

7 $$\delta = \frac{{q_{i} }}{{q_{j} }}$$

where $\delta$ is the heat radiation attenuation efficiency of the water curtain; *q*_i_ is the average heat flux at 1.5 m from the water curtain in the plane area, kW/m^2^; and *q*_j_ is the average heat flux at 2.3 m from the air curtain in the plane area, kW/m^2^. The experimental parameters were set up with different heat release rates *Q*, water curtain rows *d*, and spray pressures *p* so that the smoke blocking rate of the water curtain is primarily the function *f* (*Q*,*d*,*p*).

**Figure 13:**
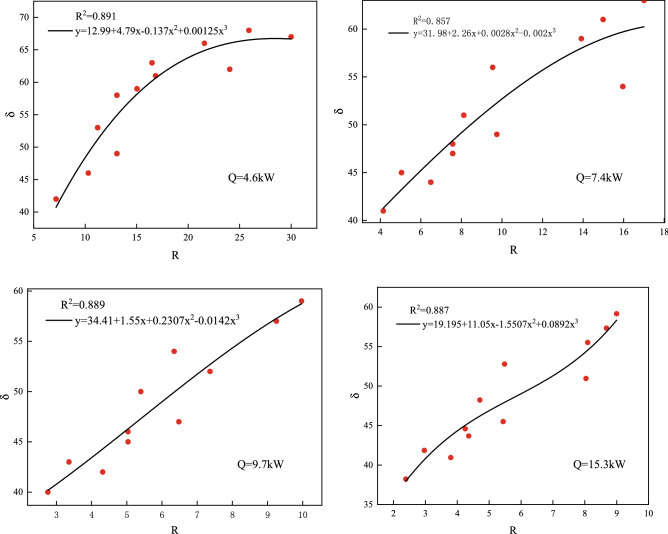
Variation curve of smoke blockage $\delta$ vs. momentum ratio *R.*

The experimental measurement points yielded 48 sets of smoke blockage $\delta$ and momentum ratios *R*, and the fitted curves were obtained by combining the smoke resistance and momentum ratios, as shown in Fig. 13.

As shown in Fig. 13, the experimentally measured smoke blockage is greater than 40%, and as the momentum ratio *R* increases, so does the overall smoke blockage, indicating a positive correlation trend. The detailed parameters are shown in Table 4. The R^2^ curve coefficients of determination are all approximately 0.865, close to 1, indicating that the fitted curve functions are highly reliable. Greater smoke blockage indicates greater heat flux attenuation, which means less heat is transmitted by the smoke, implying that the kinetic energy of the water curtain is much greater than that of the smoke. This is because the greater the rate of heat release from the fire source, the greater the kinetic energy of the smoke, and thus the smaller the resulting momentum ratio *R*, when the kinetic energy of the water curtain is maintained at a certain level.

**Table 4:** Fitting results of functions.

HRR	Fitting formula	R^2^
4.6 kW	y = 12.99 + 4.79x − 0.137x^2^ + 0.00125x^3^	0.891
7.4 kW	y = 31.98 + 2.26x + 0.0028x^2^ + 0.002x^3^	0.857
9.7 kW	y = 34.41 + 1.55x + 0.2307x^2^ − 0.0142x^3^	0.889
15.3 kW	y = 19.195 + 11.05x − 1.5507x^2^ + 0.0892x^3^	0.887

### Water curtain model for predicting heat flux in tunnel fires

#### Model analysis

The difference in heat flux before and after the water curtain, as seen from the water curtain smoke blockage equation, is a key indicator of the effectiveness of smoke blockage. The tunnel water curtain was therefore subjected to a quantitative analysis. The following formula can be obtained by assuming that the difference in heat flux before and after the water curtain *q*, and temperature T, fire heat release rate *Q*, water curtain spray pressure *p*, and water curtain width *d* are related to air density *ρ*_0_, air temperature T_0_, constant air flow pressure specific heat C_p_, and gravitational acceleration *g*.

8 $$f\left( {Q,q,p,d,\rho_{0} ,T_{0} ,C_{p} ,g} \right) = 0$$

Four dimensionless terms can be derived from this equation for dimensional analysis using the fundamental principle of dimensional analysis (the π theorem), along with earlier empirical formulas. As a power law function, the difference in heat flux *q* can be expressed as follows.

9 $$q^{*} {\text{ = k}}\left( {Q^{*} } \right)^{{{\text{k}}_{1} }} \left( {d^{*} } \right)^{{{\text{k}}_{2} }} \left( {p^{*} } \right)^{{{\text{k}}_{3} }}$$

where $q^{*} = \frac{q}{{\rho_{0} C_{P} T_{0} \sqrt {gH} }}$; $Q^{\prime\prime} = \frac{Q}{{\rho_{0} C_{P} T_{0} \sqrt {gH^{5} } }}$; d* = 0.3r; and p* = p. The coefficients k, k_1_, k_2_, and k_3_ are unknown.

#### Forecasting models

**Figure 14:**
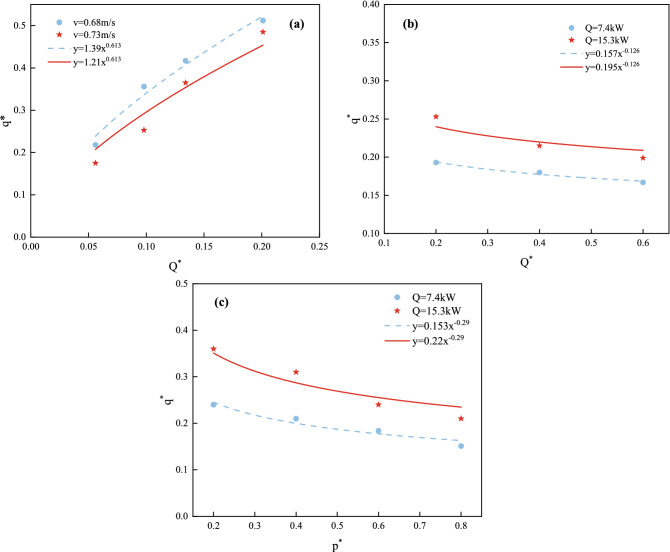
Relationship between dimensionless heat flow and dimensionless *Q*, *d*, *p*

A quantitative relationship for each dimensionless parameter was fitted using the control variables method to investigate the relationship between each dimensionless parameter and the heat flux attenuation of the water curtain; the detailed parameters are shown in Table 5, and the results of the fit are shown in Fig. 14. Figure 14a depicts the effect of different fire source heat release rates on the heat flux attenuation values of the water curtain. The dimensionless heat flux difference grows by a factor of 0.36 when compared to the dimensionless fire heat release rate, indicating that heat flux decay is positively related to the fire heat release rate. This is because the higher the kinetic energy of the hot smoke, the higher the temperature and the greater the amount of hot smoke passing through the water curtain, resulting in an increase in heat radiation behind the water curtain. The R^2^ coefficients of determination of the curves are 0.98133 and 0.9643, which are both close to 1. Fitting the results yields k_1_ = 0.36, indicating that the fitted curve function is highly reliable.

**Table 5:** Fitting results of functions.

Variable	Fitting formula	R^2^	Variable	Fitting formula	R^2^
Q* (*v* = 0.68 m/s)	y = 1.39x^0.613^	0.98133	Q* (*v* = 0.73 m/s)	y = 1.21x^0.613^	0.9643
d* (*Q* = 7.4 kW)	y = 0.157x^−0.126^	0.9914	d* (*Q* = 15.3 kW)	y = 0.195x^−0.126^	0.9472
P* (*Q* = 7.4 kW)	y = 0.153x^−0.29^	0.9844	P* (*Q* = 15.3 kW)	y = 0.22x^−0.29^	0.946

The effect of water curtain parameters on *Q* = 7.4 kW and *Q* = 15.3 kW was also investigated. Figure 14b depicts the effect of the number of water curtain rows, also known as the water curtain width, on the attenuation of heat radiation before and after the water curtain, with a curve fit of – 0.126 and coefficients of determination R^2^ of 0.9914 and 0.9472, respectively. The heat flux tends to decrease significantly as the number of water curtain rows increases, whereas a larger water curtain width increases the mass flow rate of the water curtain significantly, thus increasing the kinetic energy of the water curtain, which is more effective in controlling the diffusion of heat and smoke. Because the dimensionless q* and water curtain width are negatively correlated, the best smoke blockage effect is obtained when the water curtain width *d* is infinite. However, from an economic standpoint, it is not prudent to increase the width arbitrarily. The effect of the water curtain nozzle pressure on the heat radiation attenuation from the water curtain is shown in Fig. 14c. At a constant power generated by the fire, the velocity of the hot smoke remains constant, the kinetic energy remains constant, and the heat flux decreases with an increase in water curtain pressure, with a curve fit of − 0.29 and coefficients of determination R^2^ of 0.9844 and 0.946, respectively.

**Figure 15:**
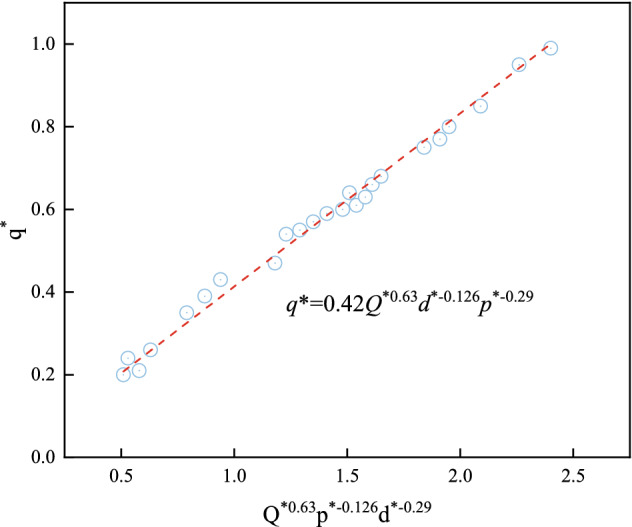
Dimensionless heat flux model test results.

Figure 15 shows the results of dimensionless heat flux model test. According to the Table 5, k1, k2, and k3 are 0.63, − 0.126, and − 0.29, respectively. To determine the coefficient k, it is also known that (Q*)^0.63^ (d*)^−0.126^ (p*)^−0.29^, so q* and k (Q*)^0.63^ (d*)^−0.126^ (p*)^−0.29^ were linearly fitted. Figure 10 depicts the experimental data for 4.6 kW and 9.8 kW fire source heat release rates to obtain the variation curves for q* and (Q*)^0.63^ (d*)^−0.126^ (p*)^−0.29^. With a coefficient of determination R^2^ of 0.9749, the results for the dimensionless thermal radiation attenuation difference fluctuate around the fitted line, and the following equation can be obtained by Eq. (9).

10 $$q^{*} { = 0}{\text{.42}}\left( {Q^{*} } \right)^{0.36} \left( {d^{*} } \right)^{{{ - }0.126}} \left( {p^{*} } \right)^{{{ - }0.29}}$$

## Conclusions

In this paper, the influence of pressure, number of rows and the power generated by the fire in a water curtain system on flue gas blockage and thermal radiation attenuation in tunnels is discussed through a reduced-size model (1:10) experiment. The experiment was carried out under longitudinal ventilation, which can avoid the phenomenon of reverse paving. The experimental data involve the temperature values extracted from the thermocouple tree at different longitudinal positions in the vertical central plane of the tunnel, which are mainly measured at two heights of the thermocouple. The main conclusions are as follows:

Adding a water curtain system has a good effect on smoke suppression and heat insulation of low-power fires in tunnels. In the overall temperature change of the tunnel, the greater the power is, the more obvious the longitudinal temperature change and the clearer the temperature bifurcation. When the water spray pressure is higher and the number of water curtain rows is greater, the water mist system will have a better smoke suppression effect. However, in the experiment, combustion smoke could not be prevented, and some smoke still penetrated the water curtain and spread.
(2)Momentum conservation through the use of dimensionless parameters *R* represents the ratio of water curtain momentum to smoke momentum, which can be a more intuitive characterization of the water curtain system's smoke blocking capacity. The larger *R* is, the stronger the water curtain system's smoke blocking capacity. The ratio of heat flux before and after the water curtain also indicates the ability of the water curtain system to block smoke; the higher the ratio $\delta$, the better the smoke blocking effect. It was discovered that when the momentum ratio *R* and the heat flux ratio $\delta$ were combined, the smoke blocking rate $\delta$ showed a positive correlation between 40 and 75% as the momentum *R* increased and the overall smoke blocking rate $\delta$ increased.
Based on the theoretical analysis and experimental results, a predictive model of water curtain attenuation heat radiation in tunnel fires is established. The quantitative relationship between the thermal radiation attenuation of the water curtain and various parameters is provided, which provides theoretical support for the quantitative study of smoke suppression and heat insulation effects of the water curtain.

## Data Availability

The datasets generated and analyzed during the current study are available from the corresponding author on reasonable request.
